# Light Influences the Growth, Pigment Synthesis, Photosynthesis Capacity, and Antioxidant Activities in *Scenedesmus falcatus*

**DOI:** 10.1155/2024/1898624

**Published:** 2024-01-23

**Authors:** Rattanaporn Songserm, Yoshitaka Nishiyama, Nuttha Sanevas

**Affiliations:** ^1^Department of Botany, Faculty of Science, Kasetsart University, Bangkean, Bangkok 10900, Thailand; ^2^Department of Biochemistry and Molecular Biology, Graduate School of Science and Engineering, Saitama University, Shimo-Okubo 255, Sakura-ku, Saitama 338-8570, Japan

## Abstract

Light plays a significant role in microalgae cultivation, significantly influencing critical parameters, including biomass production, pigment content, and the accumulation of metabolic compounds. This study was intricately designed to optimize light intensities, explicitly targeting enhancing growth, pigmentation, and antioxidative properties in the green microalga, *Scenedesmus falcatus* (KU.B1). Additionally, the study delved into the photosynthetic efficiency in light responses of *S. falcatus*. The cultivation of *S. falcatus* was conducted in TRIS-acetate-phosphate medium (TAP medium) under different light intensities of 100, 500, and 1000 *μ*mol photons m^−2^·s^−1^ within a photoperiodic cycle of 12 h of light and 12 h of dark. Results indicated a gradual increase in the growth of *S. falcatus* under high light conditions at 1000 *μ*mol photons m^−2^·s^−1^, reaching a maximum optical density of 1.33 ± 0.03 and a total chlorophyll content of 22.67 ± 0.2 *μ*g/ml at 120 h. Conversely, a slower growth rate was observed under low light at 100 *μ*mol photons m^−2^·s^−1^. However, noteworthy reductions in the maximum quantum yield (Fv/Fm) and actual quantum yield (Y(II)) were observed under 1000 *μ*mol photons m^−2^·s^−1^, reflecting a decline in algal photosynthetic efficiency. Interestingly, these changes under 1000 *μ*mol photons m^−2^·s^−1^ were concurrent with a significant accumulation of a high amount of beta-carotene (919.83 ± 26.33 mg/g sample), lutein (34.56 ± 0.19 mg/g sample), and canthaxanthin (24.00 ± 0.38 mg/g sample) within algal cells. Nevertheless, it was noted that antioxidant activities and levels of total phenolic compounds (TPCs) decreased under high light at 1000 *μ*mol photons m^−2^·s^−1^, with IC_50_ of DPPH assay recorded at 218.00 ± 4.24 and TPC at 230.83 ± 86.75 mg of GAE/g. The findings suggested that the elevated light intensity at 1000 *μ*mol photons m^−2^·s^−1^ enhanced the growth and facilitated the accumulation of valuable carotenoid pigment in *S. falcatus*, presenting potential applications in the functional food and carotenoid industry.

## 1. Introduction

Light is one of the major factors that influence algal growth, pigmentation, and photosynthesis, and light intensity can effectively regulate the metabolic induction of compounds that are valuable in responses to biochemical and physiological changes [1–3]. Microalgae perform oxygenic photosynthesis, which converts light energy into chemical energy [4]. Low light limits growth because the mutual shading of cells causes steep gradients of light, whereas high light accelerates photosynthetic electron transport, with the resultant production of reactive oxygen species (ROS) that can potentially damage cellular components, such as proteins, nucleic acids, and membrane lipids [2, 5].

Microalgae are small unicellular photosynthesis organisms that live in saline or freshwater environments. They comprise diverse groups of microorganisms of some 72,500 species that use light and carbon dioxide for growth and biomass production [4, 6–8]. Microalgae have been considered an important resource in biomass production since 1950 [9]. They are great sources of value-added products that support the nutritional and energy needs of humans, such as proteins, lipids, vitamins, and antioxidants, and also are sources of pigments. The production of biofuels using microalgae is expected to become a source of renewable energy in the future [4, 8, 10].

The investigation into the antioxidant activity of microalgae has revealed their potential as a rich source of substances with high antioxidant capacity, positioning them as advantageous natural antioxidants. Microalgae exhibit abundance in pigments, phenols, polysaccharides, proteins, essential fatty acids, vitamins, and other bioactive nutrients of high value, thereby presenting a potent repository of natural antioxidants. Notably, while the prevailing market for natural antioxidants predominantly relies on land plants, antioxidants derived from microalgae are presently not widely available [11]. Nevertheless, microalgae hold promise as a prospective source of natural antioxidant products due to their greater yield compared to terrestrial plants and the controllability of their culture conditions [12]. In the realm of microalgae, phenolic and flavonoid compounds have been identified in numerous species, including *Arthrospira maxima*, *Euglena cantabrica*, *Chlorella* sp., *Phormidium* sp., *Tetraselmis* sp., *Isochrysis* sp., and *Phaeodactylum* sp. Several reports underscore the connection between phenolic acids and antioxidant concentrations within microalgae [13]. This underscores the potential of microalgae as a prolific source of diverse bioactive compounds and a promising avenue for developing natural antioxidants, offering distinct advantages over their terrestrial counterparts.

One of the important groups of bioactive compounds produced by microalgae is carotenoids. More than 1100 carotenoids have been identified in living organisms [14–16]. Carotenoids are associated with photosynthesis in microalgae and serve to protect cells from oxidative damage and high light [15, 17]. Carotenoids also serve as commercially important nutrients in animal feed supplements, as colorants, and as compounds for cosmetic and pharmaceutical purposes [18]. Of the several microalgae that produce lutein, *Muriellopsis* sp. and *Scenedesmus almeriensis* have been tested under growth conditions for large-scale biomass production [8]. The global carotenoid market had a value of approximately 1.5 million dollars in 2017 and reached 2 billion dollars by 2022 [15].

*Scenedesmus*, which belongs to the order Chlorococcales of the family Scenedesmaceae, is frequently dominant in freshwater lakes and rivers and is commonly found in fresh and brackish waters, particularly under nutrient-rich conditions [19]. It was reported that Scenedesmaceae in Thailand comprised 35 taxa from 29 sampling sites [20]. Many species of this genus are used worldwide for various purposes due to their ability to adapt to harsh environments and grow rapidly and because they are easy to cultivate and manage. Many researchers have reported on the effects of temperature, light, and pH on the growth of *Scenedesmus* spp. and on their uses that could improve pharmaceutical and nutraceutical properties for various applications [19, 21–23]. In our previous study, it was shown that *S. falcatus* had high amounts of fatty acids, particularly in 28 compounds, and was a potentially good source of antioxidants with antidiabetic activities [24]. This research sought to examine the impacts of varying light intensities on the growth, chlorophyll fluorescence, antioxidant characteristics, and augmentation of carotenoid content, with the overarching goal of effectiveness of utilizing *S. falcatus* (KU.B1).

## 2. Materials and Methods

### 2.1. General Chemicals and Materials

The chemicals used in the study were purchased from various sources. TRIS base (H_2_NC (CH_2_OH)_3_ TRIS(hydroxymethyl)-aminomethane), MnCl_2_·4H_2_O, and acetic acid glacial were purchased from Carlo Erba (France); NH_4_Cl was purchased from Fluka (Switzerland); MgSO_4_·7H_2_O, dimethyl sulfoxide (DMSO), and Na_2_EDTA·2H_2_O were purchased from Fisher Scientific (UK); CaCl_2_·2H_2_O, K_2_HPO_4_, ZnSO_4_·7H_2_O, and CoCl_2_·6H_2_O were purchased from Ajax Finechem (Australia); KH_2_PO_4_, H_3_BO_3_, FeSO_4_·7H_2_O, CuSO_4_·5H_2_O, and sodium carbonate were purchased from Merck (Germany); (NH_4_)_6_Mo_7_O_3_ was purchased from Mallinckrodt Chemical (USA); acetone (analytical grade), methanol (analytical grade), 2,2-diphenyl-1-picrylhydrazyl (DPPH), Folin‒Ciocâlteu reagent, and gallic acid were purchased from Merck (USA); and acetone (HPLC grade) and methyl t-butyl ether (MTBE; HPLC grade) were purchased from RCI Labscan Ltd. (Thailand). Spectrophotometric determinations were performed using a UV-1800 UV-Visible spectrophotometer (Shimadzu Corp., Japan). The chlorophyll fluorescence was determined by pulse amplitude modulation (PAM-2500, Walz GmbH, Germany), and the carotenoid analysis used the high-performance liquid chromatography (HPLC) alliance e2695 separation module (Waters Corp., USA) and column C_30_ (YMC Co. Ltd., Japan).

### 2.2. Strains and Cultural Conditions

The strains of *S. falcatus* (KU.B1) were isolated and cultured in a liquid TRIS-acetate-phosphate (TAP) medium using the following method from a previous report [24]. A temperature of 25 ± 1°C and pH of 7.0 under different light intensities of 100, 500, and 1000 *μ*mol photons m^−2^·s^−1^ with a photoperiod of 12 : 12 were used.

### 2.3. Measurements of Growth

The optical density (OD) of 1 ml of the algal sample was measured at 750 nm (OD750) every 24 h up to 120 h using a UV-Visible spectrophotometer (UV-1800; Shimadzu Corporation, Japan) adapted from Chioccioli et al. [25]. The initial optical density of cell was set to 0.1 at 750 nm.

### 2.4. Measurements of Pigment

The primary pigments in algae, namely, chlorophyll a, chlorophyll b, and total carotenoids, were analyzed using a UV-Visible spectrophotometer. Harvested cultures of 5 ml were obtained at intervals of 0, 24, 48, 72, and 120 h, followed by centrifugation at 15,000 rpm for 15 min at 25°C. Subsequently, the pellet was subjected to extraction with DMSO, vortexed for 30 sec, and stored in the dark for 24 h. The supernatant obtained was spectrophotometrically measured at 480, 649, and 665 nm wavelengths. The calculation of pigment concentration was executed using the following equations [26]:

(1) $$\begin{array}{c} \text{Chlorophyll }a\;\left(\text{Chla}\right)=12.19A665-3.45A649,\\ \text{Chlorophyll }b\;\left(\text{Chlb}\right)=21.99A649-5.32A665,\\ \text{Total carotenoids}=\frac{\left[\left(1000A480\right)-\left(2.14\text{Chla}\right)-\left(70.16\text{Chlb}\right)\right]}{220}. \end{array}$$

The pigment amounts were shown in micrograms per milliliter (*μ*g·ml^−1^).

### 2.5. Measurements of Chlorophyll Fluorescence

Chlorophyll fluorescence assessments were conducted utilizing a pulse amplitude fluorometer (PAM-2500; Walz GmbH, Germany). Before measurements, algal cells were subjected to a dark acclimation period of 30 min. Subsequently, the effective quantum yield of photosystem II (YII) and the maximum quantum yield of photosystem II (Fv/Fm) were determined with an active light duration of 10 sec. Additional parameters were also quantified, including non-photochemical quenching (NPQ), photochemical quenching (qP), and relative electron transport rate (ETR).

### 2.6. Preparation of Crude Extract

A dried algal sample of 50 mg was soaked in acetone for 5 days at room temperature. The crude extract was filtered through Whatman grade 1 filter paper and evaporated using a rotary evaporator at 40°C with 250 mbar vacuum pressure and rotation at 120 rpm. The crude extract was kept at −20°C in the dark until analysis was performed.

### 2.7. Antioxidant Activity

The DPPH scavenging capacity was demonstrated using a DPPH test, according to a previously described procedure [24]. Separately, 150 *μ*l of an extract of *S. falcatus* (KU.B1) was mixed with 150 *μ*l of 0.2 mM DPPH solution in methanol (150 *μ*l). After an incubation period of 30 min at 25°C, the absorbance was measured at 520 nm.

### 2.8. Total Phenolic Compounds (TPCs)

The Folin–Ciocâlteu test was used to measure the TPC of the algal extract. This test was performed according to the method developed by Khlifi et al. [27] with some modifications. A Folin–Ciocâlteu reagent solution was initially prepared by combining 2N Folin–Ciocâlteu reagent (10 ml) with distilled water (90 ml). For the sodium carbonate solution, 7.5 g of sodium carbonate (Na_2_CO_3_) was dissolved in 100 ml of distilled water. For the assay, 25 *μ*l of *S. falcatus* (KU.B1) extract was mixed with 125 *μ*l of Folin‒Ciocâlteu reagent solution. The resulting mixture was allowed to stand at room temperature for 5 min. Subsequently, 100 *μ*l of sodium carbonate solution was added, followed by thorough mixing, and the mixture was then incubated for 60 min. The absorbance of the solution was measured at 765 nm. The TPC content of the sample was quantified in milligrams/grams of the gallic acid equivalent (GAE) by the calibration curve generated using the GAE concentration ranging from 0 to 1 mg·L^−1^: *y* = 0 = 0.2919*x* + 0.1298, *R*^2^=0.8748.

### 2.9. Analysis of Carotenoids

The crude extract was redissolved in acetone, filtered through a membrane of 0.45 *μ*m pore size, and injected in an amount of 20 *μ*l into the HPLC e2695 separation module. For the mobile phase solvent, two mobile phases was used, A and B. Phase A was methanol-MTBE-water of 81 : 15 : 4, v/v/v, and phase B was methanol-MTBE-water of 7 : 90 : 4, v/v/v with a carotenoid C_30_ column of 250 × 4.6 mm I.D. Detection was done with a PDA detector at a temperature of 25°C and an arranged flow rate of 1 ml·min^−1^. The UV wavelength scanning was 210 to 550 nm.

### 2.10. Statistical Analysis

The results were expressed as the mean of three replicated values, accompanied by the standard deviation (mean ± SD, *n* = 3). Statistical significance (*p* < 0.05) was assessed using both the analysis-of-variance (ANOVA) test and *t*-test in the context of a completely randomized design (CRD).

## 3. Results

### 3.1. Effects of Light at Various Intensities on the Growth of *S. falcatus*

**Figure 1:**
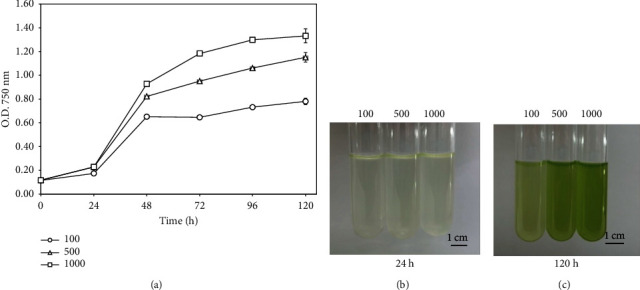
Growth of *S. falcatus* (KU.B1) cells under different light intensities of 100, 500, and 1000 *μ*mol photons m^−2^·s^−1^. Growth curves of *S. falcatus* (KU.B1) (a) and representative images of cell culture at 24 h (b) and 120 h (c). Data are means ± standard deviation (SD) (*n* = 3).

The investigation into the growth of *S. falcatus* (KU.B1) in TAP medium was conducted across varied light intensities of 100, 500, and 1000 *μ*mol photons m^−2^·s^−1^. The growth of *S. falcatus* (KU.B1) exhibited a discernible dependence on the applied light intensity. Notably, *S. falcatus* demonstrated exponential growth up to the 48 h (Figure 1(a)). The maximum optical density values of the culture under 100, 500, and 1000 *μ*mol photons m^−2^·s^−1^ at 120 h were 0.78 ± 0.06, 1.15 ± 0.04, and 1.33 ± 0.03, respectively. After 24 h of cultivation, the cell culture exposed to light exhibited a gradual increase in intensity, appearing pale green (Figure 1(b)), eventually manifesting as a green culture after 120 h (Figure 1(c)), particularly notable in the cultivation under 1000 *μ*mol photons m^−2^·s^−1^.

### 3.2. Effects of Different Light Intensities on Photosynthetic Pigments in *S. falcatus*

**Figure 2:**
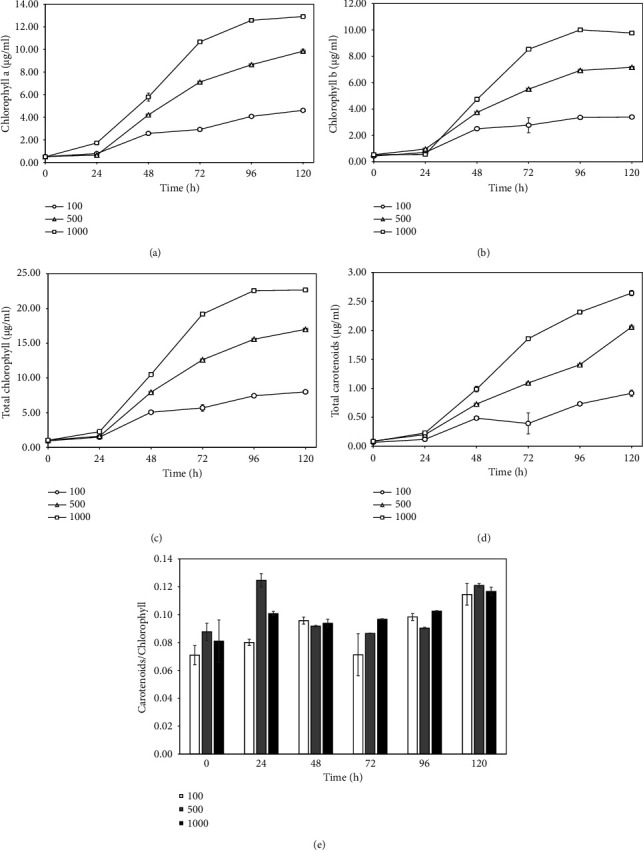
Effects of light at 100, 500, and 1000 *μ*mol photons m^−2^·s^−1^ on the amounts of chlorophylls and carotenoids in *S. falcatus* (KU.B1). Chlorophyll a (a), chlorophyll b (b), total chlorophyll (c), total carotenoids (d), and carotenoid/chlorophyll ratio (e). Data are expressed as mean ± standard deviation (SD) (*n* = 3).

The measurement of pigments, specifically chlorophyll a, chlorophyll b, and carotenoids, along with the evaluation of the carotenoid/chlorophyll ratio in *S. falcatus* (KU.B1), was conducted from the 24 h to the 120 h. The highest amounts of chlorophyll a, chlorophyll b, total chlorophyll, and carotenoids at 120 h after cultivation under light at 1000 *μ*mol photons m^−2^·s^−1^ were 12.91 ± 0.08, 9.76 ± 0.13, 22.67 ± 0.2, and 2.65 ± 0.05 *μ*g/ml, respectively (Figures 2(a)–2(d)). The ratio of carotenoids/chlorophyll, at 0.13 ± 0.01, observed at 24 h under light at 500 *μ*mol photons m^−2^·s^−1^, was slightly higher than that of 120 h (Figure 2(e)).

### 3.3. Effects of Different Light Intensities on Chlorophyll Fluorescence in *S. falcatus*

**Figure 3:**
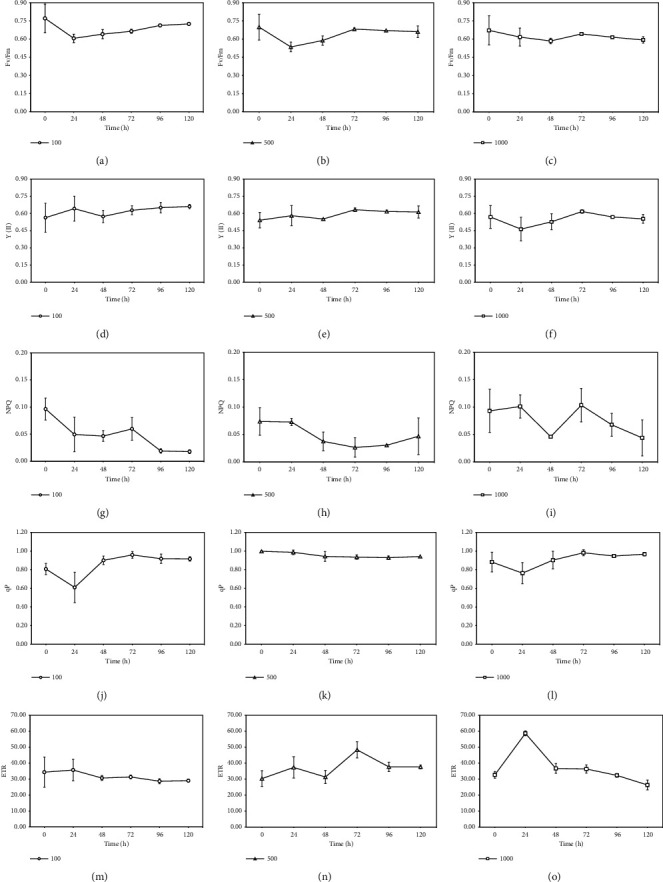
Effects of light at 100, 500, and 1000 *μ*mol photons m^−2^·s^−1^ on various chlorophyll fluorescence parameters. Maximum quantum yield (Fv/Fm) (a–c), actual quantum yield (Y(II)) (d–f), nonphotochemical quenching (NPQ) (g–i), photochemical quenching (qP) (j–l), and electron transport rate (ETR) (m–o) in *S. falcatus* (KU.B1). Data are means ± standard deviation (SD) (*n* = 3).

The photosynthetic capacity of *S. falcatus* (KU.B1) in response to light at 100, 500, and 1000 *μ*mol photons m^−2^·s^−1^ was evaluated by determining Fv/Fm, Y(II), NPQ, qP, and ETR (Figure 3).

**Figure 4:**
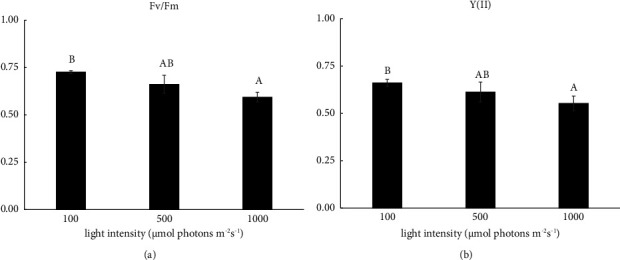
The maximum quantum yield (Fv/Fm) (a) and actual quantum yield (Y(II)) (b) under light at 100, 500, and 1000 *μ*mol photons m^−2^·s^−1^ of *S. falcatus* (KU.B1) at 120 h. Data are expressed as mean ± standard deviation (SD) (*n* = 3). Significant differences are indicated by different alphabets above the bars (a and b) at *p* < 0.05.

**Figure 5:**
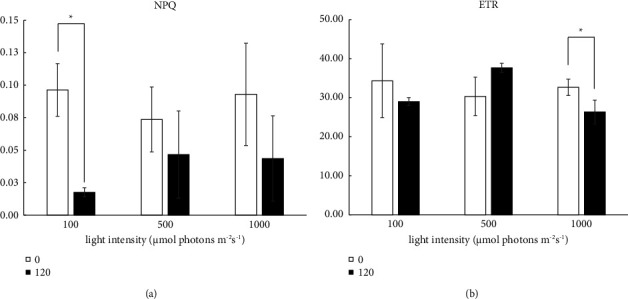
The non-photochemical quenching (NPQ) (a) and electron transport rate (ETR) (b) under light at 100, 500, and 1000 *μ*mol photons m^−2^·s^−1^ in *S. falcatus* (KU.B1) before cultivation (0 h) and after cultivation for 120 h. Data are expressed as mean ± standard deviation (SD) (*n* = 3). Statistically significant differences are denoted by asterisk above the bars at *p* < 0.05.

The Fv/Fm value ranged from 0.772 ± 0.118 to 0.606 ± 0.035 under 100 *μ*mol photons m^−2^·s^−1^, from 0.699 ± 0.207 to 0.536 ± 0.040 under 500 *μ*mol photons m^−2^·s^−1^, and from 0.672 ± 0.120 to 0.585 ± 0.023 under 1000 *μ*mol photons m^−2^·s^−1^ (Figures 3(a)–3(c)). The Y(II) value ranged from 0.564 ± 0.127 to 0.661 ± 0.018 under 100 *μ*mol photons m^−2^ s^−1^, from 0.541 ± 0.068 to 0.633 ± 0.016 under 500 *μ*mol photons m^−2^·s^−1^, and from 0.463 ± 0.153 to 0.617 ± 0.015 under 1000 *μ*mol photons m^−2^·s^−1^ (Figures 3(d)–3(f)). During cultivation under 1000 *μ*mol photons m^−2^·s^−1^ for 24 h, the Y(II) value decreased to 0.463 ± 0.153 (Figure 3(f)). The Fv/Fm and Y(II) values exhibited significant declines with increasing light intensity (Figure 4). The NPQ value significantly decreased from 0.096 ± 0.002 to 0.018 ± 0.004 at 120 h under 100 *μ*mol photons m^−2^·s^−1^ (Figures 3(g) and 5(a)). Additionally, the ETR value showed a significant decrease under 1000 *μ*mol photons m^−2^·s^−1^ (Figures 3(m)–3(o) and 5(b)). In contrast, the qP value did not significantly change even under light at 1000 *μ*mol photons m^−2^·s^−1^ (Figures 3(j)–3(l)).

### 3.4. Antioxidant Activity and Total Phenolic Compound (TPC) Assessment

To evaluate the antioxidant capacity of *S. falcatus* (KU.B1) under varying light intensities, the DPPH test was conducted. Under high light conditions at 1000 *μ*mol photons m^−2^·s^−1^, the ability to scavenge DPPH was significantly reduced with an IC_50_ of 218.00 ± 4.24 *μ*g·ml^−1^, whereas under lower light conditions at 100 *μ*mol photons m^−2^·s^−1^, the IC_50_ was 128.50 ± 7.78 *μ*g·ml^−1^ (Table 1).

The assessment of TPC in *S. falcatus* (KU.B1) using the Folin–Ciocâlteu reagent revealed variations across different light intensities. The TPC values ranged from 546 ± 58.26 mg of GAE/g under 100 *μ*mol photons m^−2^·s^−1^ to 435.33 ± 12.59 mg of GAE/g under 500 *μ*mol photons m^−2^·s^−1^ and 230.83 ± 86.75 mg of GAE/g under 1000 *μ*mol photons m^−2^·s^−1^ (Table 1). Notably, the highest TPC value was recorded under light at 100 *μ*mol photons m^−2^·s^−1^, and this value did not significantly increase under higher light intensities at 500 *μ*mol photons m^−2^·s^−1^. Additionally, the TPC content significantly decreased under 1000 *μ*mol photons m^−2^·s^−1^.

### 3.5. Individual Carotenoid Quantification

The quantification of carotenoids, including lutein, canthaxanthin, and beta-carotene, was conducted using high-performance liquid chromatography (HPLC). Notably, *S. falcatus* exhibited a higher beta-carotene production than lutein or canthaxanthin. Particularly under high light conditions at 1000 *μ*mol photons m^−2^·s^−1^, the quantities of lutein (34.56 ± 0.19 mg/g sample), canthaxanthin (24.00 ± 0.38 mg/g sample), and beta-carotene (919.83 ± 26.33 mg/g sample) significantly surpassed those observed under light intensities of 100 and 500 *μ*mol photons m^−2^·s^−1^ (Table 2).

## 4. Discussion

### 4.1. Impact of Light on Growth and Pigment Accumulation in Algae

Light serves as a fundamental resource driving microalgae growth through photosynthesis. The optimum light intensity for growth and biomass production varies across microalgae species, influenced by factors such as culture medium, temperature, light regime, and nutrient availability. However, excessive light can induce photoinhibition when it surpasses the capacity for photosynthetic reactions [7, 9, 28]. Our finding revealed that the green microalga *S. falcatus* (KU.B1) exhibited tolerance of high light intensities ranging from 500 to 1000 *μ*mol photons m^−2^·s^−1^, as evidenced by sustained exponential growth and elevated levels of photosynthetic pigments. Particularly noteworthy was the heightened growth and pigment accumulation observed in *S. falcatus* (KU.B1) under high light intensities at 1000 *μ*mol photons m^−2^·s^−1^ compared to 100 or 500 *μ*mol photons m^−2^·s^−1^. Photosynthesis is a light-dependent process, and an optimal balance of light is crucial for energy capture and conversion into biomass [29]. Microalgae employ diverse strategies to mitigate excessive light, and the observed increase in pigmentation, including chlorophylls and carotenoids, under high light conditions suggests a photoprotective response [30]. Assessing chlorophyll a, chlorophyll b, and carotenoid content in *S. falcatus* (KU.B1) provided valuable insights into the physiological responses of the microalga to different light intensities. Chlorophylls are pivotal pigments in photosynthesis, playing a crucial role in capturing light energy to synthesize organic compounds. Conversely, carotenoids contribute to light harvesting and serve as photoprotective agents [31]. The observed exponential growth of *S. falcatus* (KU.B1) under high light conditions at 1000 *μ*mol photons m^−2^·s^−1^ aligns with the elevated chlorophyll a and chlorophyll b content. This suggests that the microalga thrives under these conditions, utilizing the abundant light for efficient photosynthesis. The higher chlorophyll content likely contributes to increased light absorption and conversion capacity, promoting overall growth. A moderate growth was observed under moderate light conditions (500 *μ*mol photons m^−2^·s^−1^), accompanied by a proportional chlorophyll content. This indicates that *S. falcatus* (KU.B1) can adapt to moderate light intensities, albeit with a growth and intermediate chlorophyll synthesis between low and high light conditions. The slower growth observed under low light conditions (100 *μ*mol photons m^−2^·s^−1^) correlates with a decreased chlorophyll content. *S. falcatus* (KU.B1) appears light-limited under these conditions, leading to a suboptimal photosynthetic rate and overall growth inhibition. The substantial increase in carotenoid content under high-light conditions suggests a photoprotective response. Carotenoids are crucial in dissipating excess light energy and protecting the photosynthetic apparatus from potential damage. The heightened carotenoid accumulation under 1000 *μ*mol photons m^−2^·s^−1^ indicates an adaptive mechanism of *S. falcatus* (KU.B1) to efficiently manage the intense light environment.

Comparable light intensity effect has been observed in other microalgal species, including *Desmodesmus quadricauda*, *Parachlorella kessleri*, and *Chlamydomonas reinhardtii*, each exhibiting optimal growth within specific light intensity ranging from 100 to 500 *μ*mol photons m^−2^·s^−1^ [4]. Our results align with the previous studies on *Scenedesmus* green microalgae (i.e., *Scenedesmus* sp. ADIITEC-II and GUBIOTJ116, *S. obliquus*, and *S. quadricauda*), which displayed optimal growth rates and biomass production under light intensities of 81, 150, and 500 *μ*mol photons m^−2^·s^−1^, respectively [21, 32]. Importantly, the response to high light at 1000 *μ*mol photons m^−2^·s^−1^ inhibited the growth of *S. quadricauda*, while low light at 100 *μ*mol photons m^−2^·s^−1^ impeded algal cell growth [9]. These observations underscore the species-specific nature of optimal light intensities for microalgae cultivation.

### 4.2. Chlorophyll Fluorescence

In addition to its impact on algal cell growth and photosynthetic pigment levels, light intensity variations significantly influenced photosynthetic efficiency, particularly concerning photosystem II (PSII) activity. Chlorophyll fluorescence provides an informative tool to assess the effects of environmental stresses on plants, offering parameters such as Fv/Fm, Y(II), NPQ, qP, and ETR to quantify PSII efficiency and assess photosynthetic performance [33]. In our study, the response of *S. falcatus* (KU.B1) to varying light conditions revealed that high light intensity at 1000 *μ*mol photons m^−2^·s^−1^ led to the reduction in the Fv/Fm ratio and Y(II) value. This decline in photosynthetic efficiency suggests a mechanism of inhibitory effects induced by high light stress. The decrease in efficiency may arise from either damage to PSII, where the photodamage rate surpasses the repair rate, or initiation of NPQ processes [34–36]. This aligns with observations in other microalgae species, such as *Dunaliella salina*, which showed a reduction in the Fv/Fm ratio under higher light conditions exceeding 500 *μ*mol photons m^−2^·s^−1^, indicative of photoinhibition [36]. Similarly, in the case of *Arthrospira platensis*, a reduction in the Fv/Fm ratio during midday was attributed to intensified solar radiation due to heightened light conditions [37]. Furthermore, observations made on *Alaria esculenta* and *Muriellopsis* sp. indicated a decrease in the Fv/Fm ratio when exposed to high light conditions, consistent with our results [8, 38]. However, in our results, the photosynthesis continuously occurred under the high light intensity of 1000 *μ*mol photons m^−2^·s^−1^. This intriguing observation implies that *S. falcatus* (KU.B1) may have developed an efficient repair cycle, enabling rapid turnover of damaged PSII components in response to heightened light intensity and maintaining optimal photosynthetic efficiency [36].

NPQ provides insights into the intra-thylakoid pH gradient and the chloroplasts' capacity to disperse excessive excitation energy as heat, which corresponds linearly to energy dissipation, effectively protecting cells from photodamage [36, 39]. Our investigation revealed that *S. falcatus* (KU.B1) cultivated under high light at 1000 *μ*mol photons m^−2^·s^−1^ exhibited minimal NPQ induction, suggesting that *S. falcatus* (KU.B1) may have efficiently adapted to manage the intense light environment possibly due to the accumulation of substantial carotenoid quantities. Carotenoids contribute to NPQ by dissipating excess energy as heat [40]. The ability to control NPQ may help the alga balance light absorption and utilization for photosynthesis. In contrast with outcomes from a study involving *Phaeodactylum tricornutum*, exposure to high light at 280 *μ*mol photons m^−2^·s^−1^ resulted in a noticeable NPQ decline [41].

The qP represents the proportion of light energy trapped by PSII reaction centers for electron transport [42]. In our result, qP exhibited a slight decrease at the low light conditions (100 *μ*mol photons m^−2^·s^−1^) and high light conditions (1000 *μ*mol photons m^−2^·s^−1^) during a 24 h cultivation period. Previous studies on *D. salina* exposed to light conditions at 200, 500, and 1000 *μ*mol photons m^−2^·s^−1^ showed no fluctuations in qP, while a slight decrease was observed when cells were cultured under 1500 *μ*mol photons m^−2^·s^−1^ [36].

The relative electron transport rate (ETR) stands as a pivotal parameter, offering insights into the photosynthetic efficiency of algae across varied light conditions [43, 44]. In our study, under high light conditions of 1000 *μ*mol photons m^−2^·s^−1^, a significant reduction in ETR was observed in *S. falcatus* (KU.B1) after 120 h. The decrease in ETR under high light conditions suggests that, despite the microalga thriving and exhibiting exponential growth under these intense luminous conditions, there is a limit to the photosynthetic capacity. High light intensities can lead to photoinhibition, a process where the rate of photodamage to the photosystem exceeds the repair rate, resulting in a decline in photosynthetic efficiency [45]. The observed reduction in ETR may indicate a protective mechanism employed by *S. falcatus* (KU.B1) to mitigate the potential damage caused by excessive light. Microalgae often deploy various strategies to cope with high light stress, including thermal dissipation of excess energy (NPQ) and adjustments in the electron transport chain to optimize energy use [36, 39]. The decrease in ETR might be a regulatory response to prevent overexcitation of the photosynthetic apparatus and to maintain a balance between energy absorption and utilization. Interestingly, the ability of *S. falcatus* (KU.B1) to exhibit sustained growth under high light conditions, despite the reduction in ETR, suggests that this microalga possesses adaptive mechanisms to acclimate to elevated light intensities efficiently. *S. falcatus* (KU.B1) may have evolved into a dynamic photosynthetic apparatus, effectively managing and distributing absorbed light energy, even under stressful conditions. This observation also aligns with findings from *Chlamydopodium fusiforme*, wherein a decreased ETR was noted during a four-day outdoor cultivation period [46].

### 4.3. Antioxidants and TPC

The DPPH assay evaluates the antioxidant capacity of tested compounds by measuring their ability to reduce DPPH radicals through direct electron transfer or radical quenching involving H atom transfer [47]. Our results revealed a significantly elevated DPPH scavenging activity in *S. falcatus* (KU.B1) under optimal growth conditions with low and moderate light intensities (100 and 500 *μ*mol photons m^−2^·s^−1^, respectively). Conversely, under high light intensity of 1000 *μ*mol photons m^−2^·s^−1^, the DPPH scavenging activity was notably diminished. Elevated light intensity has the potential to induce oxidative stress in algae. Although algae produce antioxidants as a defense mechanism against oxidative stress, excessively high light conditions may surpass the antioxidant defense capacity, reducing scavenging activity [48]. Excessive light can induce photoinhibition, a process where the photosynthetic apparatus is damaged, affecting the overall metabolic processes. This damage could decrease the production or effectiveness of antioxidant compounds, leading to reduced DPPH scavenging activity [49]. Furthermore, high-light conditions might trigger a shift in the metabolic pathways of algae. Some pathways related to antioxidant production could be downregulated, or the energy resources could be redirected to cope with other stress responses, consequently impacting DPPH scavenging [50]. Mishra et al. [51] reported enhanced growth along with increased amounts of carbohydrates, carotenoids, lipids, and antioxidative activity in *Isochrysis galbana* under high light at 325 *μ*mol photons m^−2^·s^−1^. Furthermore, Dinev et al. [52] studied extracts from *S. dimorphus* and found that a high antioxidant potential determined by the DPPH method correlated with elevated levels of total phenolic and flavonoid compounds. Increased phenolic production serves as a direct countermeasure against free radicals, interrupting the sequence of reactions in lipid peroxidation chain reactions [53]. Consistently, our study demonstrated enhanced antioxidant efficacy from low light at 100 *μ*mol photons m^−2^·s^−1^ to high light at 500 *μ*mol photons m^−2^·s^−1^, along with increased TPC content.

### 4.4. Individual Carotenoid Content

Light intensity significantly influences the quantity and composition of carotenoids, playing a pivotal role in the substantial accumulation of various carotenoids, including beta-carotene, lutein, and astaxanthin, within microalgae. This influence demonstrates distinctive traits specific to each species [17, 54]. Our results revealed high light-stimulated carotenoid synthesis in *S. falcatus* (KU.B1), dominantly with beta-carotene, lutein, and canthaxanthin, respectively. Carotenoids are essential pigments involved in photosynthesis. They capture light energy and transfer it to chlorophyll, enhancing light absorption efficiency and utilization in the photosynthetic process [55]. One of the primary functions of carotenoids is to protect the photosynthetic apparatus from damage caused by excess light energy. They act as antioxidants, scavenging reactive oxygen species generated during photosynthesis and preventing oxidative stress [45]. Algae often adjust the composition and concentration of carotenoids in response to changes in light conditions. Under high light intensity, algae may increase carotenoid production to enhance photoprotection, while they may reduce carotenoid synthesis under low light conditions. This is particularly important during stressful conditions, such as exposure to high light or environmental fluctuations. Under elevated light conditions, *Scenedesmus* sp. KGU-Y002 accumulated total carotenoids at 34.2 ± 3.8 mg·g^−1^ dry weight per cell [56]. In our study, beta-carotene consistently manifested as the preeminent carotenoid across all examined light intensities, followed by lutein. Earlier studies have outlined prevalent carotenoids in green algae, including beta-carotene, lutein, violaxanthin, and zeaxanthin. Notably, these carotenoids demonstrate a more extensive distribution among green algal species than higher plants [57]. Lutein and beta-carotene are classified as primary carotenoids and play crucial roles in photosynthesis within the chloroplast [58]. Beta-carotene, a primary microalgal carotenoid that transfers light energy to chlorophylls and mitigates oxidative damage in microalgae [16], accumulates in *D. salina* in response to increased light exposure [59]. Microalgae, under suitable culture conditions, can accumulate lutein. For instance, under high light, microalgae utilize lutein to reduce oxidative damage through NPQ. *Scenedesmus* sp. accumulated approximately 7.47 mg/g dry weight or 19.70 mg/l/day of lutein when cultivated under 400 *μ*mol photons m^−2^·s^−1^ [60]. Canthaxanthin, a secondary carotenoid, also shows light intensity-dependent accumulation [61]. *S. obliquus,* a green alga, accumulates significant canthaxanthin [62]. In the green alga *Coelastrella striolata* var. *multistriata*, the content of canthaxanthin, among a mixture of carotenoids, reached 47.5 mg/g dry weight at its maximum, depending on the light intensity [63]. Exploring various species, Coulombier et al. [54] reported that *Nephroselmis* sp., *Dunaliella* sp., *Picochlorum* sp., *Nitzschia* sp. A, and *Entomoneis punctulata* exhibit elevated total carotenoid and individual carotenoid contents under high light conditions (600 *μ*mol photons m^−2^·s^−1^). Conversely, *Tetraselmis* sp., *Schizochlamydella* sp., *Nitzschia* sp. B, *Thalassiosira weissflogii*, *Cylindrotheca closterium*, and *Chaetoceros* sp. demonstrate heightened total carotenoid content under a low light intensity **(**250 *μ*mol photons m^−2^·s^−1^). The discernible impact of light intensity appears to be distinctly species-specific.

## 5. Conclusions

Our investigation underscores the profound influence of light intensity on the cultivation dynamics of *Scenedesmus falcatus* (KU.B1), influencing key parameters such as biomass production, pigment content, and metabolic compound accumulation. This comprehensive study aimed to optimize light conditions, specifically targeting the enhancement of growth, pigmentation, and antioxidative properties in *S. falcatus* (KU.B1). Cultivation under varying light intensities of 100, 500, and 1000 *μ*mol photons m^−2^·s^−1^ revealed a notable increase in *S. falcatus* (KU.B1) growth under high light conditions at 1000 *μ*mol photons m^−2^·s^−1^, reaching a maximum optical density of 1.33 ± 0.03 and a total chlorophyll content of 22.67 ± 0.2 *μ*g/ml at 120 h. Conversely, a slower growth was observed under low light at 100 *μ*mol photons m^−2^·s^−1^. However, the enhanced growth under 1000 *μ*mol photons m^−2^·s^−1^ was accompanied by reductions in maximum quantum yield (Fv/Fm) and actual quantum yield (Y(II)), indicating a decline in photosynthetic efficiency. Notably, the high light conditions at 1000 *μ*mol photons m^−2^·s^−1^ led to a significant accumulation of beta-carotene, lutein, and canthaxanthin within algal cells, showcasing the potential for valuable carotenoid pigment production. This suggests promising applications in the functional food and carotenoid industries. However, despite the positive impact on pigmentation and growth, antioxidant activities and total phenolic compounds decreased under high light conditions at 1000 *μ*mol photons m^−2^·s^−1^. The observed trade-off between enhanced growth and decreased antioxidative properties under high light conditions underscores the need for a balanced approach in optimizing light parameters for microalgae cultivation. Future studies could delve deeper into the intricate mechanisms governing this trade-off and explore strategies to mitigate potential oxidative stress while maximizing pigment production. Overall, these findings contribute valuable insights into optimizing light conditions for microalgae cultivation, emphasizing the potential for *S. falcatus* (KU.B1) in applications related to biomass production and the carotenoid industry.

## Figures and Tables

**Table 1:** Antioxidant activity and total phenolic compound (TPC) in *S. falcatus* (KU.B1) under light at 100, 500, and 1000 *μ*mol photons m^−2^·s^−1^.

Light intensity (*μ*mol photons m^−2^·s^−1^)	DPPH (IC_50_)	TPC (mg of GAE/g)
100	128.50 ± 7.78^a^	546.00 ± 58.26^b^
500	147.00 ± 7.07^a^	435.33 ± 12.59^b^
1000	218.00 ± 4.24^b^	230.83 ± 86.75^a^

Data are presented as mean ± standard deviation (SD) (*n* = 3). Different alphabets indicate significant differences (a–b) within the same column at *p*  <  0.05.

**Table 2:** Carotenoid content in *S. falcatus* (KU.B1) grown under light at 100, 500, and 1000 *μ*mol photons m^−2^·s^−1^.

Light intensity (*μ*mol photons m^−2^·s^−1^)	Lutein (mg/g sample)	Canthaxanthin (mg/g sample)	Beta-carotene (mg/g sample)
100	23.20 ± 0.61^a^	10.38 ± 0.19^a^	799.17 ± 22.73^b^
500	25.97 ± 0.42^b^	19.92 ± 0.23^b^	599.47 ± 2.00^a^
1000	34.56 ± 0.19^c^	24.00 ± 0.38^c^	919.83 ± 26.33^c^

Data are presented as mean ± standard deviation (SD) (*n* = 3). Different alphabets indicate significant differences (a–c) within the same column at *p*  <  0.05.

## Data Availability

The data used to support the findings of this study are included within the article.
